# Revisiting gametocyte biology in malaria parasites

**DOI:** 10.1093/femsre/fuz010

**Published:** 2019-04-09

**Authors:** Priscilla Ngotho, Alexandra Blancke Soares, Franziska Hentzschel, Fiona Achcar, Lucia Bertuccini, Matthias Marti

**Affiliations:** Wellcome Centre for Integrative Parasitology, Institute of Infection, Immunity and Inflammation, University of Glasgow, 120 University Road, Glasgow G12 8TA, UK; Wellcome Centre for Integrative Parasitology, Institute of Infection, Immunity and Inflammation, University of Glasgow, 120 University Road, Glasgow G12 8TA, UK; Wellcome Centre for Integrative Parasitology, Institute of Infection, Immunity and Inflammation, University of Glasgow, 120 University Road, Glasgow G12 8TA, UK; Wellcome Centre for Integrative Parasitology, Institute of Infection, Immunity and Inflammation, University of Glasgow, 120 University Road, Glasgow G12 8TA, UK; Core Facilities, Microscopy Area, Instituto Superiore di Sanita, Via Regina Elena 299, 00161 Rome, Italy; Wellcome Centre for Integrative Parasitology, Institute of Infection, Immunity and Inflammation, University of Glasgow, 120 University Road, Glasgow G12 8TA, UK; Department of Immunology and Infectious Diseases, Harvard T.H. Chan School of Public Health, Boston 02115, MA, USA

**Keywords:** *Plasmodium falciparum*, gametocyte, malaria, transmission

## Abstract

Gametocytes are the only form of the malaria parasite that is transmissible to the mosquito vector. They are present at low levels in blood circulation and significant knowledge gaps exist in their biology. Recent reductions in the global malaria burden have brought the possibility of elimination and eradication, with renewed focus on malaria transmission biology as a basis for interventions. This review discusses recent insights into gametocyte biology in the major human malaria parasite, *Plasmodium falciparum* and related species.

## Teaser

This review provides an update on our current understanding of the only parasite stage that can transmit malaria from man to mosquito, the gametocyte.

## INTRODUCTION

Over the past decade, mass roll-outs of effective control tools for malaria have resulted in a significant reduction of malaria disease and death, as well as transmission rates in most endemic countries. Recently, this decline has stalled in many of these countries, and in some, a reversal of these gains is observed (WHO 2018). Importantly, the frontline antimalarials do not effectively clear gametocytes from infected people emphasising their unique biology compared to the asexual blood stage parasites (Delves *et al*. 2016; Bradley *et al*. 2019). Resurgence of human malaria and emergence of zoonotic infections emphasize the critical role of transmission for the persistence and spread of malaria in humans. Our knowledge about parasite transmission biology is mostly based on *Plasmodium falciparum* and the rodent malaria model *Plasmodium berghei*. By comparison, less research has been done on other parasites including *Plasmodium vivax* and *Plasmodium knowlesi*. However, major advances have been made in the past few years in our understanding of parasite transmission biology. Here, we discuss these recent findings with a focus on the only stage capable of transmission to mosquitoes, the gametocyte.

### *Plasmodium* species relevant for human disease or research

Malaria parasites infect a variety of vertebrate hosts from mammals to birds and reptiles. The 6 human infective *Plasmodium* species of public health importance are *P. falciparum*, *P. vivax*, *P. malariae*, *P. ovale curtisi*, *P. ovale wallikeri* and *P. knowlesi*. Although *P. falciparum* malaria is the most lethal form that causes the bulk of morbidity and mortality, *P. vivax* malaria is the most prevalent form of disease outside of Africa and will persist once *P. falciparum* has been eliminated. In the past decade, *P. knowlesi* has emerged as a significant source of zoonotic infections in Southeast Asia (Singh and Daneshvar 2013), probably through increased contact of humans with the natural host and vector system. In addition to the canonical human malaria species, *Plasmodium cynomolgi* has been reported to infect humans asymptomatically in western Cambodia (Imwong *et al*. 2019) and *P. brasilianum* in the Venezuelan Amazon (Lalremruata *et al*. 2015). *Plasmodium* cyno*molgi* has been used as an *in vivo* model for *P. vivax* in non-human primates. Similarly, *P. knowlesi* has been established as an *in vitro* model for *P. vivax*. The most popular *in vivo* malaria models are, however, rodent malaria parasites used for different aspects of disease and immunological studies: *P. berghei* is the most studied of these parasites, as it can also be used to induce experimental cerebral malaria, thus modelling an important clinical complication of *P. falciparum* infection. The *P. berghei* model is also characterised by the ability to very efficiently generate all parasite life stages under laboratory conditions and its ease of genetic modification. It has therefore been very useful to elucidate many aspects of parasite infection *in vivo* including mosquito transmission. Recently *P. falciparum* gametocyte development has been established in the humanised mouse model as a system to study some features of gametocytogenesis *in vivo* (Duffier *et al*. 2016).

### Phylogeny of the *Plasmodium* genus

**Figure 1.**
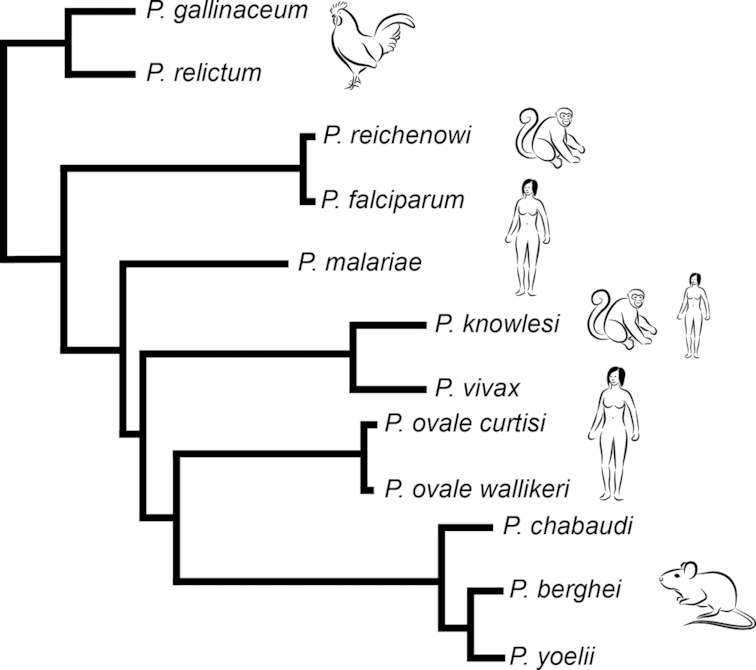
Phylogenetic representation of *Plasmodium* lineages. Schematic of phylogenetic tree based on most recent genome information across *Plasmodium* lineage. The tree is adapted from the recent publication by Boehme *et al*. (2018). The data show the major *Plasmodium* lineages: bird (*P. gallinaceum*, *P. relictum*) parasites are at the base of the tree whilst *Laverania* (including *P. falciparum* and *P. reichenowi*) are a sister group to other *Plasmodium* within the clade of mammalian parasites. The latter include rodent malaria parasites (*P. berghei*, *P. chabaudi*, *P. yoelii*) as well as primate (*P. knowlesi*) and human parasites (*P. ovale curtisi* and *P. ovale wallikeri*, *P. vivax* and*P. malariae*).

Over the past decade, major genome sequencing projects have been completed providing a large library of reference genomes from different *Plasmodium* species across the major lineages (e.g. Gardner *et al*. 2002; Carlton *et al*. 2008; Otto *et al*. 2014; Rutledge *et al*. 2017). Subsequent comparative genomic analyses have started to clarify the evolutionary relationships between *Plasmodium* species. The first such analysis was done between the *P. falciparum* whole genome and *P. yoelii yoelii* partial genome, revealing a high level of synteny across the two species especially in the central chromosome regions (Carlton *et al*. 2002). The most recent phylogenomic analysis across *Plasmodium* confirms the ancient branching of bird and reptile parasites compared to all parasites infecting mammals (Bohme *et al*. 2018). In this analysis, rodent malaria parasites form a sister clade with the primate parasite lineage (represented by *P. knowlesi* and *P. vivax*) and the *P. ovale* lineage, whilst *Laverania* (the group of primate parasites including *P. falciparum*) and *P. malariae* are placed closer to the base (Fig. 1). Interestingly, a recent genetic analysis (analyzing 21 genetic loci) of the sister lineages *Plasmodium* and *Haemoproteus* including a large number of parasites infecting bats suggests that *Plasmodium* is polyphyletic and forms several independent clades (*Laverania*, rodent parasites, bird and reptile parasites, primate parasites and *P. malariae*/*P. ovale*) within the *Haemoproteus* lineage (Galen *et al*. 2018). These studies highlight the complicated evolutionary past of malaria parasites and suggest switches between and adaptations to host and vector. This is of particular relevance for parasite transmission, as some of the key aspects of this process appear to differ between *Plasmodium* lineages, although the overall structure of the life cycle remains the same.

### The *Plasmodium* life cycle

All malaria parasites leave the insect host through an infective bite by which they enter the vertebrate host and initiate the pre-erythrocytic developmental stage. In all mammalian species, this pre-erythrocytic stage occurs within the liver, where parasites invade hepatocytes, develop within the parasitophorous vacuole (PV) and produce tens of thousands of red blood cell (RBC) infective merozoites in a process called schizogony. Some species, such as *P. vivax*, *P. ovale* and *P. cynomolgi*, produce a latent phase (hypnozoite) during the pre-erythrocytic stage that remains in hepatocytes for prolonged durations (up to years). Notably, avian malaria parasites make hypnozoites during both the pre-erythrocytic cycle and in the erythrocytic cycle (Valkiunas 2005). Latent stages can be activated and initiate an infection, complicating *P. vivax* control strategies. From the pre-erythrocytic cycle, RBC-infective merozoites emerge and initiate the blood stage (erythrocytic) cycle of the parasite. After invasion of RBCs, these merozoites develop within a PV and undergo schizogony to produce daughter merozoites, which burst out of the host cell and reinvade new RBCs to perpetuate this asexual replication cycle. This asexual cycle causes all the symptoms associated with malaria infection and it can result in RBC infection rates >10% in malaria patients. The length of the intraerythrocytic cycle and the number of merozoites generated is species-specific. In some species, including *P. berghei* and *P. vivax*, merozoites preferentially invade young erythrocytes, increasing the likelihood of anemia (Cromer *et al*. 2006; Malleret *et al*. 2015).

A subset of asexually replicating parasites will produce gametocyte progeny and initiate the sexual cycle. Gametocytes develop in the intermediate vertebrate host until maturation before they are taken up by an arthropod. A series of recent studies suggest that in some species only mature gametocyte stages are present in the blood circulation, whilst immature gametocytes sequester in host tissues, particularly bone marrow and spleen (Joice *et al*. 2014; De Niz *et al*. 2018; Lee, Waters and Brewer 2018; Obaldia *et al*. 2018). Once ingested by the vector, the sexual reproduction phase of the life cycle occurs within the insect's midgut, allowing for recombination and generation of variation within the parasite population. During gametogenesis, the male gametocyte divides into up to eight flagellated microgametes, whereas the female gametocyte develops into a single macrogamete. Fertilization of a macrogamete by a microgamete results in the formation of a zygote that undergoes meiosis and develops into an ookinete, a motile form with apical organelles. The ookinete penetrates the mosquito gut wall and comes to rest near the basal lamina of the midgut. Here the ookinete rounds up and transforms into an oocyst, within which the parasite asexually replicates, forming several thousand sporozoites (sporogony). Upon oocyst rupture, these sporozoites migrate to and invade the salivary glands, where they can be transmitted back to the vertebrate host during a blood meal.

### Phases of the gametocyte developmental cycle

**Figure 2.**
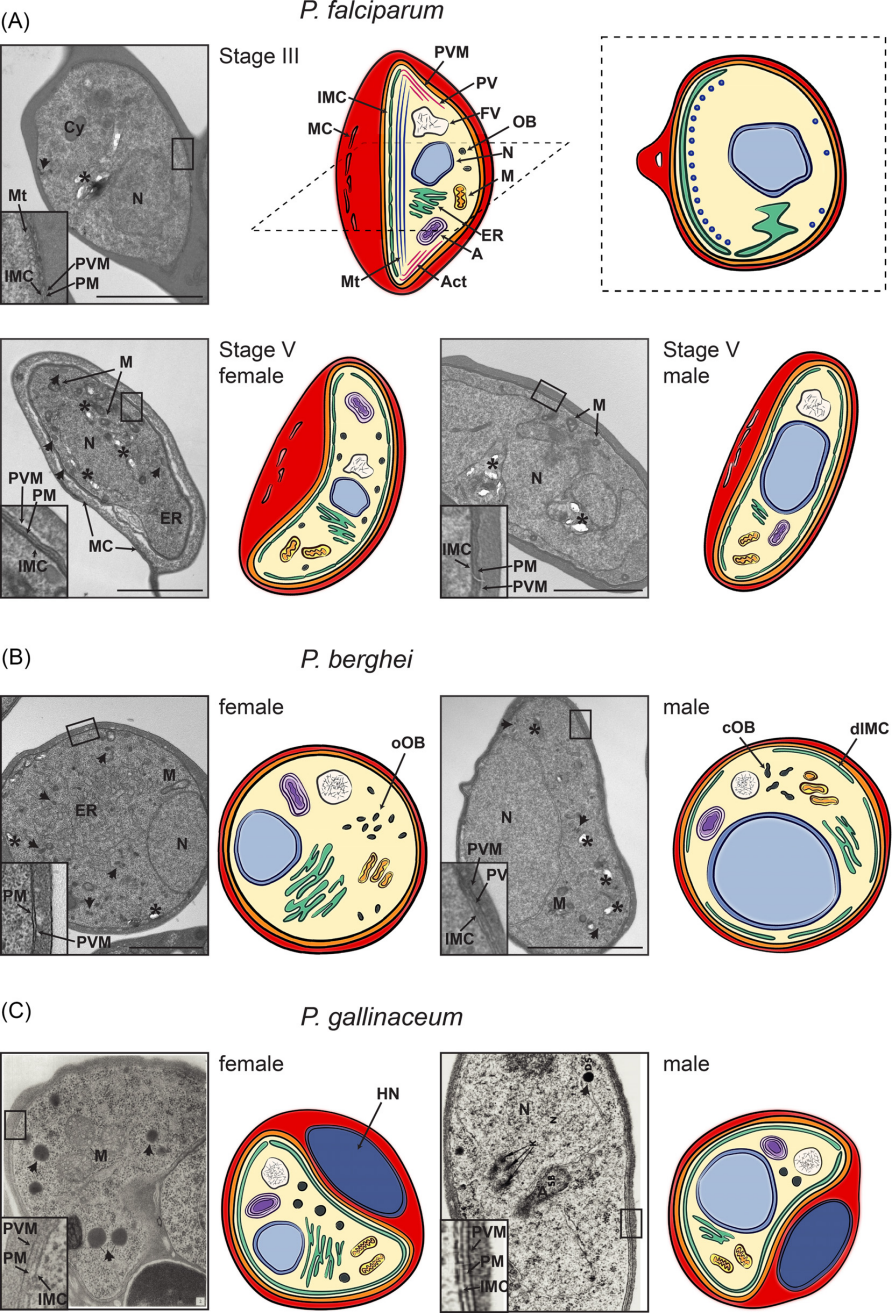
Morphological features of gametocytes across *Plasmodium* lineages. Shown are representative electron microscopy images and drawings of gametocytes of (**A**) human (*P. falciparum*), (**B**) rodent (*P. berghei*) and (**C**) avian (*P. gallinaceum*) malaria parasites. Mitochondria (M), apicoplast (A), nucleus (N), endoplasmatic reticulum (ER) and the food vacuole (FV) containing hemozoin crystals (marked with an asterisk) are found in all *Plasmodium* species in both male and female gametocytes. The gametocyte plasma membrane (PM) is in close association with the parasitophorous vacuolar membrane (PVM), and in most cases with the IMC (depicted in the closeup insets of the electron micrographs). Whilst the nucleus is smaller and more compact in female compared to male gametocytes, the ER is more developed in female gametocytes, in line with increased translation. Osmiophilic bodies (OB, marked with an arrow) are found in all species, with a higher number in female than in male gametocytes. (**A**) Ultrastructure of immature stage III and mature stage V *P. falciparum* gametocytes. Upper panel: Electron micrograph (left) and drawing (middle: longitudinal section, right: cross-section) of a stage III gametocyte. The IMC develops along one side of the gametocyte, supported by underlying microtubules (Mt)(depicted in the inset). Some microtubules are also found on the opposing side (see cross-section). Additionally, actin filaments (Act) are found, in particular at the tips of the developing gametocyte. MC are found within the erythrocyte cytoplasm. Lower panel: Electron microscope image and drawing of a female (left) and male (right) stage V gametocyte. The IMC plates now completely surround the gametocytes whilst actin and microtubules are absent. In the female gametocyte osmiophilic bodies are located along the periphery of the cell. In contrast they have not been described in male *P. falciparum* gametocytes. (**B**) Electron micrograph and drawing of mature female (left) and male (right) *P. berghei* gametocytes. Osmiophilic bodies are oval (oOB) and more abundant in female gametocytes, whilst male gametocytes have fewer and club-shaped osmiophilic bodies (cOB). The IMC is difficult to observe in female gametocytes and possibly absent, whilst a discontinuous IMC (dIMC) is clearly detectable in male *P. berghei* gametocytes (see insets) (Mons 1986). (**C**) Electron micrograph and scheme of mature female (left) and male (right) *P. gallinaceum* gametocytes. Electron micrographs are taken from (Aikawa *et al*. 1969)(female) and (Sterling and Aikawa 1973)(male) [PERMISSION PENDING]. In contrast to mammalian mature RBCs, avian mature RBCs are nucleated (depicted as host nucleus (HN)).

Most parts of the *Plasmodium* transmission cycle are conserved across the genus (Fig. 2); however, there are notable differences in cycle length and morphology between lineages. The conserved features enable utilization of animal models for *in vivo* studies of transmission biology. The first developmental stage that is functionally different from an asexual parasite is the sexually committed schizont. Commitment to gametocytogenesis is initiated by the activation of the transcription factor AP2-G, both in rodent parasites and *P. falciparum* (Kafsack *et al*. 2014; Sinha *et al*. 2014). Consistent with a previous report (Bruce *et al*. 1990), a recent study has demonstrated that *P. falciparum* schizonts produce either asexually or sexually committed progeny (Brancucci *et al*. 2018). Interestingly, conditional activation of AP2-G can produce mixed progeny, both in *P. falciparum* and *P. berghei* (Bancells *et al*. 2019; Kent *et al*. 2018). Upon invasion, the earliest phases of gametocyte development are morphologically indistinguishable from asexual development. Subsequently, the gametocytes of *P. falciparum* undergo five morphologically discernible stages of development in the course of 9–12 days (Hawking, Wilson and Gammage 1971). Stage I gametocytes have a very similar appearance as asexual trophozoites and cannot be distinguished morphologically; however, they can be identified using genetic reporters, as their transcriptome begins to differ from the one of asexual parasites. In stage II, slight changes in appearance become apparent, as the parasite takes the shape of a lemon or oat grain with one pointed end. The parasite then elongates whilst one side flattens and the opposite membrane curves, so that in stage III the shape of the parasite resembles the letter ‘D’ (Fig. 2A). The length of the parasite exceeds the diameter of the RBC, which exhibits signs of deformation. Developing into stage IV, the parasite elongates even more, now resembling the shape of a banana. The RBC cytoplasm is almost completely occupied with the parasite, except for a small membrane fold known as the Laveran's bib. Stage V gametocytes exhibit the characteristic crescent shape with rounded ends, as opposed to the pointed ends of stage IV gametocytes (Sinden 1982)(Fig. 2A). Apart from the likely conservation in *Laverania*, the morphological features of *P. falciparum* gametocytes are not conserved across the *Plasmodium* lineage: most gametocytes from rodent, bird and primate parasites are round and resemble the trophozoite stage (Sinden *et al*. 1978; Walzer *et al*. 2018)(Fig. 2B and C). The 9–12 day maturation time for *P. falciparum* gametocytes is exceptionally long, whereas the development in other primate, avian and rodent *Plasmodium* species ranges from 24 to 60 hours (Gautret and Motard 1999). In addition following development, and in contrast to species with shorter developmental periods that survive for less than 12 hours in circulation, mature *P. falciparum* gametocytes can survive for several days before being taken up by a mosquito (Smalley and Sinden 1977; Gautret and Motard 1999; Eichner *et al*. 2001).

### Regulation of gametocytogenesis

Sexual commitment is a facultative step in the malaria parasite cycle, and the rate of commitment is responsive to environmental cues. Similarly, gametocyte activation and subsequent gametogenesis depend on environmental cues presented upon parasite ingestion by the mosquito. Therefore, various steps in the transmission cycle are highly regulated and finely tuned at multiple levels. Gametocytes represent only a small fraction of the parasite population both *in vitro* and during infection, and rates of commitment vary (see above), representing a major barrier for their systematic investigation. Several environmental factors, including drugs, host cells and extracellular vesicles have been proposed to lead to sexual commitment of asexual blood stage parasites (Josling, Williamson and Llinas 2018). Whilst stage-specific effects of certain drugs may result in an increased sexual conversion rate, recent data do not support a physiological role for host cell age or extracellular vesicles in commitment (Brancucci *et al*. 2017). In contrast, a class of phospholipids abundant in serum, lysophosphatidylcholines (LysoPC), has been identified as sufficient to repress the gametocyte commitment at physiological levels in *P. falciparum* (Brancucci *et al*. 2017). LysoPC breakdown products are used for phospholipid biosynthesis via the Kennedy pathway in the parasite and blocking this pathway can induce sexual commitment (Brancucci *et al*. 2018). Altogether these data strongly suggest that the abundance of environmental sensors regulates both parasite metabolism and sexual commitment.

Parasites adapt to new host environments in mammalian host and arthropod vector in order to optimise the balance between persistence and transmission. Recent studies have shown that different *Plasmodium* species are able to detect fluctuations in nutrient availability and modify gene expression accordingly. In *P. falciparum*, limited LysoPC availability is translated into changes in rates of growth and sexual reproduction, whilst glucose sensing only affects growth. In rodent malaria parasites, both LysoPC and glucose sensing are only linked to growth but not transmission (Brancucci *et al*. 2017; Mancio-Silva *et al*. 2017), suggesting that there are significant differences in how these two parasite lineages respond to environmental changes. Notably, a recent study has also shown that *P. falciparum* parasites produce a higher proportion of gametocytes in low transmission settings and more asexual progeny in high transmission settings (Rono *et al*. 2018). These studies demonstrate that there are epigenetic and genetic differences between and within parasite species that result in distinct responses to environmental cues, as has been demonstrated for the variant expression of components of the *Plasmodium* surface anion channel (PSAC) on the iRBC surface (Nguitragool *et al*. 2011). It is likely that genetic differences also account for the adaptation of gametocytes in different host environments; however, systematic investigation of such phenotypes is currently lacking.

### Genetics of gametocyte development

**Figure 3.**
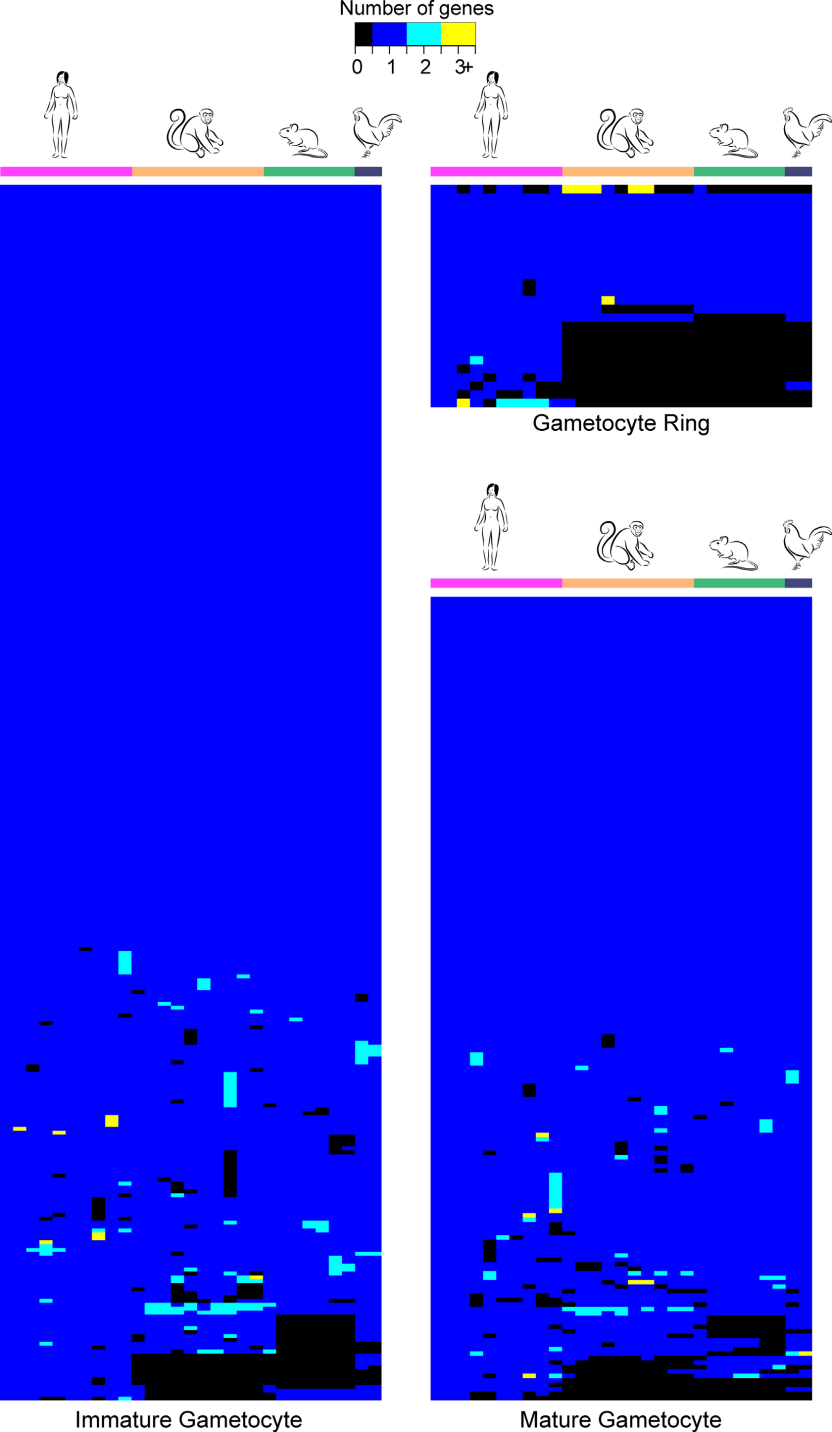
Comparative genomic view of gametocytogenesis across *Plasmodium* lineages. Heatmap showing the number of orthologous genes annotated as expressed specifically in *P. falciparum* gametocytes as defined by Pelle *et al*. (2015)(in rows) in major *Plasmodium* lineages. Apart from *P. falciparum* we have included *P. vivax*, *P. berghei* and *P. gallinaceum*. The orthologues of the *P. falciparum* gametocyte genes were retrieved from PlasmoDB (Aurrecoechea *et al*. 2009). The genes (rows) were reordered by hierarchical clustering using the R function hclust (www.R-project.org). Using *P. falciparum* as a template (as there are no other species-specific sets of gametocyte genes) the majority of gametocyte-specific genes is conserved across the 4 species (see also Supplementary Table S1 (Supporting Information)). Most *P. falciparum*-specific genes in the gametocyte ring encode exported proteins. Interestingly, a putative zinc finger protein (PF3D7_1 134 600) has been lost in the rodent lineage only. Only a few immature and mature gametocyte genes are unique to *P. falciparum*, and most of them encode hypotheticals. Amongst the few exceptions are two putative protein kinases that have been selectively lost in primate (PF3D7_031 1400) and rodent malaria parasites (PF3D7_020 3100), respectively.

Initial studies of the genetics of gametocytogenesis relied on parasites which have lost the ability to produce gametocytes when maintained in *in vitro* culture. Indeed, gene loss or amplification can occur during culture adaptation, in particular if resulting in a fitness advantage such as loss of gametocyte production. Several *P. berghei* (Sinha *et al*. 2014) and *P. falciparum* (Alano *et al*. 1995; Eksi *et al*. 2012) parasite lines have lost the ability to form gametocytes during continuous blood passage in the mouse (*P. berghei*) and *in vitro* (*P. falciparum*) due to loss of specific chromosomal loci. Importantly, whole genome sequencing identified mutations in the *ap2-g* locus in most instances (Kafsack *et al*. 2014; Sinha *et al*. 2014; Claessens *et al*. 2017), demonstrating that loss of AP2-G function results in a fitness advantage under optimised *in vitro* conditions and during propagation in the mouse. Further functional studies identified AP2-G as the key switch initiating the sexual commitment process in both *P. falciparum* and *P. berghei* (Kafsack *et al*. 2014; Sinha *et al*. 2014). Intriguingly, two recent studies using inducible expression systems have demonstrated that conditional *ap2-g* activation in ring stage parasites alone results in reprogramming of these cells into gametocytes (Bancells *et al*. 2019; Kent *et al*. 2018), without passing through the committed schizont stage. The physiological relevance of this alternative sexual commitment process is unclear. Another gene, *gdv-1* has been identified via genome sequencing of a gametocyte-deficient *P. falciparum* line (Eksi *et al*. 2012). Recent data suggest that GDV-1, possibly regulated by its own anti-sense transcript is essential for activating AP2-G (Filarsky *et al*. 2018), moving the unknown trigger further upstream. Interestingly, the *gdv-1* locus has been lost in the rodent malaria lineage (Brancucci *et al*. 2017)(see also Fig. 3), suggesting that alternative pathways of the AP2-G activation may be available in this lineage. It is currently unknown how LysoPC levels translate into *gdv-1* and *ap2-g* activation and, hence, what the upstream trigger regulating commitment is.

Additional loci have been identified that are commonly lost in long term culturing experiments, but their putative role in gametocytogenesis remains to be investigated (Claessens *et al*. 2017). An initial study exploiting the *piggybac* transposon system in *P. falciparum* enabled identification of additional genes important for formation and development of gametocytes (Ikadai *et al*. 2013). Amongst parasite mutants with defective gametocyte production, 16 unique loci were identified–9 genes interfered with formation of gametocytes and 7 with gametocyte development. More recently, a genome-wide knock-out study in *P. berghei* (Bushell *et al*. 2017) and saturation mutagenesis using *piggybac* in *P. falciparum* (Zhang *et al*. 2018) have provided a first map of essential genes in *Plasmodium*. As expected, the proportion of essential genes is significantly lower amongst gametocyte genes compared to the rest of the genome (our own analysis; *P*value < 1.5e-15, one-tailed Pearson's χ^2^). In contrast to the recent genetic dissection of sexual commitment, very little progress has been made to systematically investigate genetic determinants of gametocyte development. Most of the genes identified as selectively expressed during gametocyte development in *P. falciparum* are conserved between *Plasmodium* species (Fig. 3). A small subset of these genes appears to be lineage specific: genes specific to the *Laverania* subgenus expressed in the gametocyte ring stage are mainly annotated as exported proteins (see Supplementary Table S1, Supporting Information), whilst the function of most of the other lineage-specific genes is still unknown.

### Gene regulation during gametocytogenesis

Early studies have suggested that in *Plasmodium* gene expression is a tightly regulated cascade such that a gene is expressed just in time when the protein is required (Bozdech *et al*. 2003). This paradigm has meanwhilst been corroborated through identification and functional investigation of a family of transcription factors of the ApiAP-2 family (Coulson, Hall and Ouzounis 2004), including AP2-G. Moreover, a series of studies has demonstrated that many processes in response to environmental changes, such as antigenic variation, invasion switching and sexual commitment are also under strong epigenetic control (e.g. Stubbs *et al*. 2005; Voss *et al*. 2006; Brancucci *et al*. 2014; Coleman *et al*. 2014; Fraschka *et al*. 2018). For example, in most *Plasmodium* species studied so far, virulence genes are localised in the subtelomeric regions and in a few chromosome internal clusters, tracking with the genomic distribution of heterochromatin patterns (Fraschka *et al*. 2018). A few additional loci are similarly heterochromatic in asexual parasites and the only one conserved across *Plasmodium* encodes *ap2-g* (Fraschka *et al*. 2018), supporting its essential role in sexual commitment across species and suggesting that epigenetic regulation of this master switch is highly conserved. A series of single cell transcriptomics studies provided a first glimpse into the transcriptome of asexually and sexually committed schizonts (Poran *et al*. 2017; Brancucci *et al*. 2018). Apart from *ap2-g*, these studies identified a number of merozoite antigens with unique expression in sexually committed schizonts, including MSRP1 and DBLMSP2. These data suggest that the sexual merozoites membrane may be functionally distinct from their asexual counterparts. It is tempting to speculate that such differences relate to altered tissue or host cell tropism between asexual and gametocyte stages. Indeed, both *P. berghei* and *P. falciparum* gametocytes preferentially develop in the extravascular space of the spleen and bone marrow, whilst asexual stages do not show such a preference (Joice *et al*. 2014; De Niz *et al*. 2018; Lee, Waters and Brewer 2018).

**Figure 4.**
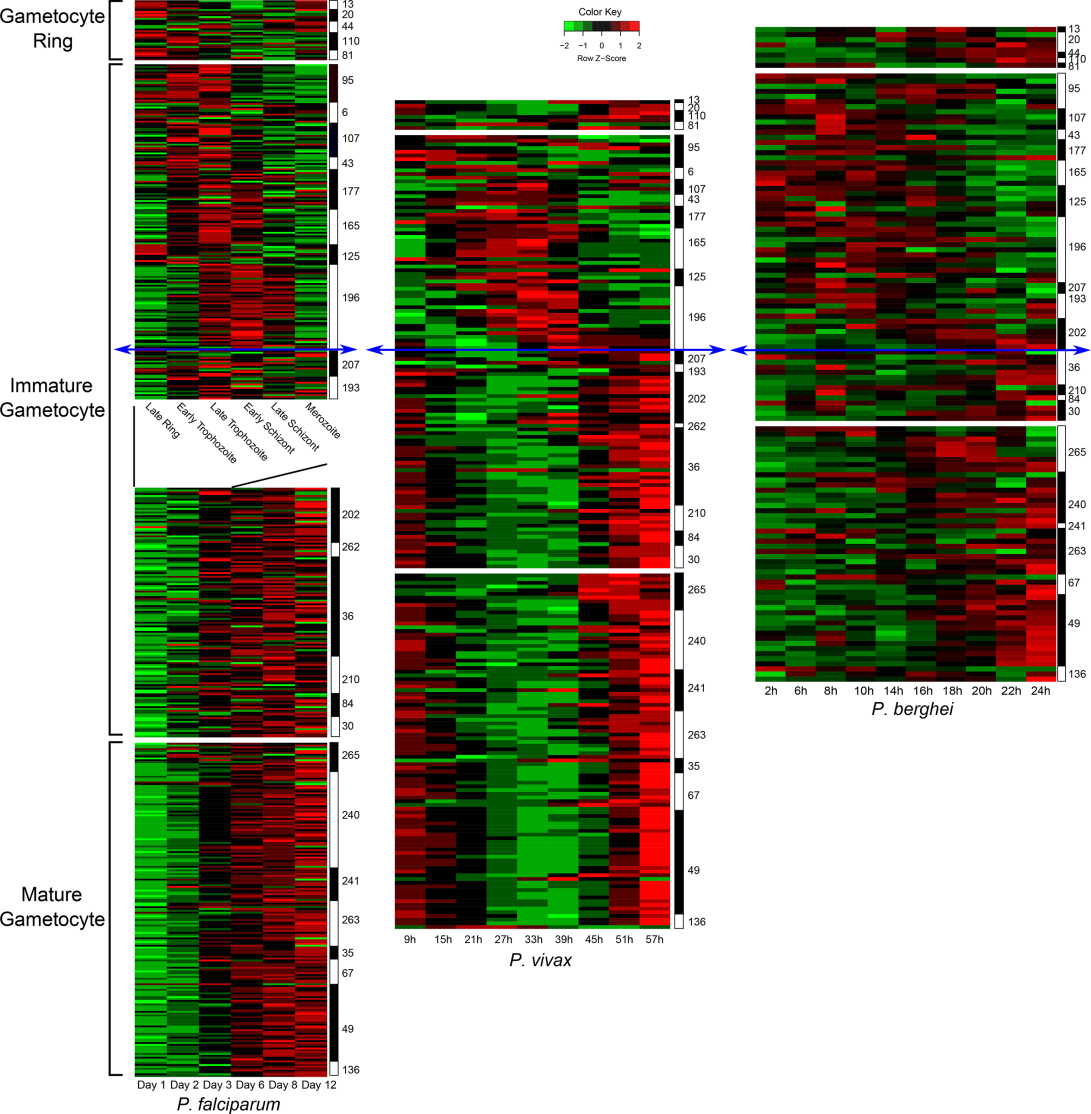
Transcriptional profiles across gametocyte development and *Plasmodium* lineages. Time-course data (Log2 Fold change) of gametocyte-specific genes were extracted from previously published datasets. For *P. falciparum* data are used from an asexual time course (Le Roch *et al*. 2003)(top part, in hours) and a gametocyte time course (Young *et al*.^2005^)(bottom part, in days). For *P. vivax*, data are used from an *ex vivo* time course (Bozdech *et al*. 2008)(in hours), and for *P. berghei* data are from an *in vivo* time course (Hoo *et al*. 2016)(in hours). Comparing the temporal transcription of the gametocyte-specific genes as defined in Pelle *et al*. and their orthologues in *P. berghei* and *P. vivax* shows that their profile is qualitatively conserved. The initial phase of development from the gametocyte ring stage to early immature gametocyte development shows a similar cascade of gene expression and takes a similar amount of time across the three species (20–39 hours). In contrast further gametocyte maturation lasts significantly longer in *P. falciparum* (9–10 days) than in *P. vivax* (about 17–20 hours) and *P. berghei* (about 4 hours). These differences in maturation time are likely related to the physiological differences in their respective hosts. The numbers on the right side indicate the cluster number as defined previously (Pelle *et al*. 2015). The blue arrows separate the time courses between the initial stages of development (top) with conserved duration and the later stages with variable in time scale.

Activation of *ap2-g* expression in a subset of schizonts triggers expression of a series of early gametocyte genes including *Pfs16*, *Pfg27/25*, *Pfg14.744*, *Pfg14.745* and *Pfg14.748* (Kafsack *et al*. 2014) that are needed in the gametocyte progeny. Many of the genes activated in response to AP2-G are required for early gametocyte development, including several members of the PHIST family (Eksi *et al*. 2005). In contrast, genes involved in asexual host cell remodeling, including components of the knob complex required for antigen display on the iRBC surface are epigenetically silenced during sexual stages (Fraschka *et al*. 2018). As in the asexual cycle, it is anticipated that gene expression during gametocyte development is tightly synchronised and coordinated. Notably, many factors required for parasite development in the mosquito stage are transcribed in gametocytes and the mRNAs are stored via translational repression (Mair *et al*. 2006; Guerreiro *et al*. 2014), providing an additional level of expression regulation. Unfortunately, the only *P. falciparum* time course data available are more than a decade old and the samples used contain mixtures of asexual and gametocyte stages (Eksi *et al*. 2005; Young *et al*. 2005). Nevertheless, one of the time courses was used, in conjunction with a large array of expression data across the entire parasite cycle, to deconvolve these mixtures by generating a transcriptional network of *P. falciparum* gene expression (Pelle *et al*. 2015)(Fig. 4). The network predicts 29 expression clusters peaking at different phases during the gametocyte cycle and probably provides the most accurate analysis of transcriptional dynamics in gametocytes to date. Most recently, both transcriptional and proteomic data were combined to generate a short list of high confidence gametocyte markers in *P. falciparum* (Meerstein-Kessel *et al*. 2018). Also, artificial *ap2-g* activation and reprogramming within the same cycle was used for transcriptional profiling of gametocyte development in *P. berghei* (Kent *et al*. 2018). Altogether these data demonstrate that virulence and transmission traits are tightly controlled and interlinked in *Plasmodium*.

### Metabolic adaptations during gametocytogenesis

Most metabolic pathways are similar between parasite and its host, but there are key differences in the enzymes involved. *Plasmodium* possesses most pathways for *de novo* biosynthesis of important metabolites (curiously, several have been lost in the rodent malaria lineage (Frech and Chen 2013)) but heavily relies on salvaging nutrients from host cells. As the parasite encounters different environments within and between hosts, it must cope with the different physiological environments in the various tissues and hosts. Nutrient sensing thereby links parasite metabolism (e.g. salvage vs*de novo* biosynthesis) to cell fate (e.g. replication vs differentiation). For example, replicating parasites have different requirements for energy metabolism than gametocytes, and the two stages may develop in different environmental niches and host cells.

Proliferative parasites rely on glycolysis to sustain high energy requirements for generating large numbers of progeny. However, *Plasmodium* parasites are unable to generate glucose or catabolise glycogen and, therefore, they rely on glucose from the host cells. Within the vertebrate host blood glucose levels are maintained at constant rate and provide the parasite a stable source for glycolysis. In turn parasites produce lactic acid as a byproduct, which can cause lactic acidosis during malaria infection. *Plasmodium* also lack the gene encoding the mitochondrial pyruvate dehydrogenase; however, both asexual stages and gametocytes catabolise both glucose and glutamine in a canonical TCA cycle for energy production (MacRae *et al*. 2013). Whilst asexual parasites rely mostly on glucose uptake and aerobic glycolysis for energy, gametocytes mostly use the mitochondrial TCA cycle for energy production (MacRae *et al*. 2013), probably due to their sequestration outside of the blood stream. It is confirmed by the fact that the deletion of the enzymes of this cycle allows for asexual replication but tends to be deleterious for gametocyte formation (Ke *et al*. 2015). Interestingly, African *Trypanosomes* show a similar pattern of energy usage: proliferating slender forms perform glycolysis whilst non-proliferating stumpy forms required for transmission use the TCA (Giffin and McCann 1989). Gametocytes possess larger mitochondria with tubular cristae (Okamoto *et al*. 2009), and a majority of mitochondrial TCA cycle enzymes are upregulated in gametocytes (Khan *et al*. 2005; Young *et al*. 2005). Male gametes have no mitochondria (Okamoto *et al*. 2009) and utilize glycolysis for motility (Talman *et al*. 2014), whilst female gametes as well as ookinetes maintain TCA use. Of note, some *Plasmodium* parasites including *P. vivax* and to a lesser extent *P. berghei* are restricted to reticulocytes that are metabolically active and have a functional TCA cycle maintained by glutamine as the main carbon source (Srivastava *et al*. 2017). Because of their enrichment in hematopoietic niches, where erythroid cells are predominant, gametocytes can potentially exploit carbon reserves in these cells in addition to utilisation of TCA for energy metabolism.

Lipids are essential in *Plasmodium* for signaling, protein trafficking, hemoglobin degradation and membrane biosynthesis. Lipid metabolism is an essential process of the parasite, which involves uptake from host cells, transport and synthesis from fatty acids (FA). *Plasmodium* parasites are capable of obtaining FA from host plasma for lipid synthesis (Grellier *et al*. 1991; Ofulla *et al*. 1993). Alternatively they can synthesise them *de novo* using a (type II) fatty acid biosynthesis pathway (Tarun, Vaughan and Kappe 2009). This process is dispensable in asexual blood stages but crucial in mosquito and liver stages (Yu *et al*. 2008; Vaughan *et al*. 2009; van Schaijk *et al*. 2014), whilst there are currently no data for gametocytes. However, there are significant differences in the lipid composition of asexual stages and gametocytes (Gulati *et al*. 2015). Overall phospholipid and glycerolipid levels are decreased in gametocyte stages, probably as a result of altered substrate utilization following the switch to gametocytogenesis (Brancucci *et al*. 2017). Gametocytes may require less lipids for membrane biosynthesis compared to the proliferative asexual stages. Interestingly, sphingolipids, and in particular ceramide levels, are increased in gametocytes and essential for their maturation (Gulati *et al*. 2015; Tran *et al*. 2016).

### Emerging roles for IMC and cytoskeleton during gametocyte development

A common feature of the Alveolates—which include the Dinoflagellates, Ciliates and Apicomplexa—are the alveoli, flattened membrane sacs beneath the plasma membrane (Gould *et al*. 2008). In Apicomplexa, the alveoli are usually referred to as inner membrane complex (IMC). Together with the plasma membrane, alveoli form the so-called pellicle, a structure that is closely associated with cytoskeletal elements. The most conspicuous function of the pellicle is to generate and maintain cell shape, such as funnel shaped Tintinnids in Ciliates, crescent shaped sporozoites in *Plasmodium*, and the horned appearance of the Ceratium species (Dinoflagellates). Furthermore, the pellicle serves as an anchor and scaffold for various parasite structures. For example, gliding motility—a hallmark of apicomplexan locomotion required for various migration processes—is facilitated by the glideosome, consisting of an actin-myosin motor complex anchored to the IMC and the plasma membrane (Baum *et al*. 2006). The pellicle also harbours the apical complex that Apicomplexan parasites require for host cell invasion and in some cases egress. In *P. falciparum* parasites, the IMC is only present in merozoites, sporozoites, ookinetes and gametocytes. Whilst merozoites, sporozoites and ookinetes actively invade or traverse host cells, and sporozoites and ookinetes exhibit gliding motility, active motility or invasion has not been conclusively shown in gametocytes. Thus, the IMC in this stage seems to predominantly play a structural role; however, recent studies imply that motility might be a feature of gametocytes as well (De Niz *et al*. 2018).

### IMC and microtubule dynamics during gametocyte development

In contrast to gametocytes, merozoites, sporozoites and ookinetes possess a polar ring at the apical end that functions as a microtubule organising centre (MTOC). In these stages, microtubules originate at the polar ring and grow towards the basal end of the cell, whilst at the same time IMC membranes spread over the cell body, finally engulfing it. In *P. falciparum* gametocytes IMC assembly is initiated by unknown factors and starts in stage I/II gametocytes with the deposition of a single patch of vacuolar membrane and microtubules underneath the parasite membrane (Sinden 1982; Dearnley *et al*. 2012; Dearnley *et al*. 2016)(Fig. 2A). Additional membrane sacs, originated from the endoplasmic reticulum (ER), are deposited at the periphery along one side of the developing gametocyte in a string of beads pattern. This side will form the ‘foot’, the flattened membrane of the stage III gametocyte. Even though gametocytes, as opposed to the motile stages of *P. falciparum*, do not possess an apical-basal polarity (Parkyn Schneider *et al*. 2017), they are clearly laterally polarised during their development. Whether this polarisation has any functional consequences, however, remains unknown.

Microtubules are present underneath the membrane sacs, indicating that they nucleate and polymerise at the developing IMC membranes. However, it is also possible that microtubule polymerisation is mediated by unknown factors in these regions and triggers or attracts the deposition of IMC membranes (Parkyn Schneider *et al*. 2017). Further experiments are needed to test this hypothesis, especially live cell imaging that facilitates a better temporal resolution of cellular processes. Whilst microtubule bundles are concentrated along the nascent IMC plates, they also start extending through the cytoplasm in early stage II gametocytes. Further microtubule polymerisation accompanied by the extension of the IMC plates then leads to the elongation of the gametocyte ‘foot’, giving it the characteristic D-shape of stage III gametocytes. In stage III gametocytes, the IMC plates start to extend laterally to finally engulf the gametocyte almost entirely in stage IV. During the lateral extension of the IMC, the division of the IMC into individual plates becomes very apparent. Proteinaceous sutures connect the plates that are approximately 800 nm wide. The sutures start forming as soon as IMC plates are deposited in stage I-II gametocytes (Kono *et al*. 2012). Apart from connecting the IMC plates, the sutures are believed to play a role in the parallel alignment of microtubules along the longitudinal axis of the gametocyte, as is apparent in stage IV gametocytes (Meszoely *et al*. 1987; Kaidoh *et al*. 1993). In stage V gametocytes, the microtubule network is disassembled and only patches of short microtubule bundles remain that are associated with the IMC (Parkyn Schneider *et al*. 2017). This leads to rounding up of the gametocyte tips and a relaxation of the IMC membranes, where the sutures are now less pronounced. The disassembly of the microtubule network coincides with a steep increase in cellular deformability compared to stage IV and younger gametocytes (Aingaran *et al*. 2012; Dearnley *et al*. 2012; Tiburcio *et al*.2012a). However, it has recently been shown that microtubules are not the only, or not even the major structural determinant to gametocyte cellular rigidity. Instead, rearrangements of the RBC cytoskeleton play a dominant role in the deformability switch (Dearnley *et al*. 2016). Functionally, this high deformability allows mature gametocytes to leave their site of sequestration in the bone marrow or spleen and to enter the circulation to be taken up by a mosquito, as shown in the case of *P. berghei* (De Niz *et al*. 2018).

Whilst IMC development in gametocytes has been extensively studied in *P. falciparum*, less is known about it in other *Plasmodium* species. Based on early electron microscopy studies, it appears that the gametocytes of most *Plasmodium* species, including avian and reptilian malaria parasites, possess an IMC (Aikawa, Huff and Sprinz 1969). However, some species, e.g. *P. berghei* (Olivieri *et al*. 2015) and *P. knowlesi* (Aikawa, Huff and Sprinz 1969) exhibit discontinuities within the IMC, i.e. the IMC does not completely engulf the gametocyte (see also Fig. 2B). Interestingly, in *P. berghei* the discontinuous IMC is easily detected in male gametocytes, whilst in female gametocytes it may be absent (Mons 1986). As the main function of the IMC in gametocytes seems to be forming and stabilising the shape of the cell, *Plasmodium* species with round gametocytes might have less requirement for a stable IMC. Further systematic ultrastructural and light microscopy studies of different *Plasmodium* species will be required to learn more about the IMC and its function(s) in gametocytes.

### Different roles for actin in gametocytes?

*Plasmodium* expresses two distinct actin isoforms, Actin-I and Actin-II. Actin-I is expressed throughout the whole life cycle whilst Actin-II is only expressed in the mosquito stages, gametocytes (mainly males) and gametes (Wesseling *et al*. 1989). A recent study has investigated Actin-I localisation through gametocyte development in *P. falciparum*. It was shown that actin forms filaments that are present underneath the microtubules and can be closely associated with them. These filaments appear very stable as they cannot be depolymerised using the actin polymerisation inhibitor Cytochalasin D. Throughout gametocyte development, actin accumulated at the opposing ends of the cell (Fig. 2A) and co-localised with the actin nucleation factor formin-1, indicating that these are the sites of actin polymerisation. In contrast to the actin filaments associated with microtubules, these filaments are sensitive to treatment with Cytochalasin D. The function of the actin network in gametocytes is not fully understood, but it appears that it is required for maintaining a normal mitochondrial architecture (Hliscs *et al*. 2015) and for successful release of male gametes (Deligianni *et al*. 2011). Another potential function could be the stabilisation of the cell, thus contributing to the high rigidity of developing gametocytes: actin filaments, similar to microtubules are disassembled in stage V gametocytes. Interestingly, treatment of *P. berghei* gametocytes with Cytochalasin D affects homing to bone marrow and spleen upon infection, suggesting that actin function is required for parasite circulation and tissue homing (De Niz *et al*. 2018).

### Extreme makeover: host cell remodelling during gametocyte development

Protein export and the modification of the host RBC are hallmarks of *Plasmodium* blood stage infection and conserved across the genus, but they have hitherto been most extensively studied in *P. falciparum*. The discovery of an N-terminal motif termed PEXEL or HT motif (Hiller *et al*. 2004; Marti *et al*. 2004) required for protein export has facilitated the prediction of the *P. falciparum* exportome. Including exported proteins without a PEXEL motif (termed PEXEL-negative exported proteins, PNEPs), 5–10% of the *P. falciparum* proteome is estimated to be exported (Spielmann and Gilberger 2015). Such a high fraction of exported proteins seems to be an exception amongst *Plasmodium* species. *Plasmodium falciparum* was found to have largely expanded its repertoire of exported PEXEL proteins compared to other human and rodent malaria species. Remarkably, the *P. falciparum* genome both encodes more exported protein families than other species and the families that are shared between species contain more members in *P. falciparum* (Sargeant *et al*. 2006). Whilst the number of PEXEL proteins indicates that *P. falciparum* indeed has a much larger repertoire of exported proteins than most other *Plasmodium* species, it is also possible that a majority of the exportome of those other species consists of PNEPs. However, the lack of a known export motif within PNEPs and their small number has made the prediction of complete exportomes so far impossible (Gruring *et al*. 2012; Heiber *et al*. 2013; Spielmann and Gilberger 2015).

### Defining the Plasmodium Gexportome

Whilst protein export and host cell modifications are essential for the asexual parasite's survival both *in vivo* and *in vitro*, less is known in the sexual stages. A comparative proteome analysis of *P. falciparum* gametocytes vs asexual trophozoites revealed an upregulation of a number of exported proteins during the early phases of gametocytogenesis (Stage I/II) (Silvestrini *et al*. 2010). These proteins, termed GEXPs (*P. falciparum* gametocyte-exported proteins) represent one-tenth of the entire proteome detected in this study, indicating that protein export plays an important role during early gametocytogenesis. Interestingly, only four of the 26 GEXPs have orthologues in *P. berghei*, indicating that the cellular processes GEXPs are involved in during early *P. falciparum* gametocytogenesis differ significantly from *P. berghei* (Silvestrini *et al*. 2010). Remarkably, one-third of the GEXPs belong to the PHIST (*Plasmodium* helical interspersed subtelomeric) protein family (Sargeant *et al*. 2006), a *Plasmodium*-specific protein family with important roles in host cell remodelling and protein trafficking (reviewed in Warncke, Vakonakis and Beck (2016)). In contrast, components of the asexual knob complex and its major surface antigen PfEMP1 are absent in *P. falciparum* gametocytes (Tiburcio *et al*. 2012b; Fraschka *et al*. 2018), suggesting that there are significant differences in terms of host cell remodelling between asexual and gametocyte stages.

Already during RBC invasion, the parasite modifies its host cell by secreting proteins and lipids into the RBC, including the proposed PSAC component CLAG3, that is essential for nutrient uptake (Nguitragool *et al*. 2011). When invasion is complete, the parasite is engulfed by a parasitophorous vacuole membrane, creating a barrier between the parasite and its host cell (Dvorak *et al*. 1975). For proteins to reach the host cell and subsequent instalment of host cell modifications, these proteins have to cross the PVM. This is achieved by inserting a protein translocon, the *Plasmodium* translocon of exported proteins (PTEX) into the PVM membrane (de Koning-Ward *et al*. 2009). It has been shown that both PEXEL proteins and PNEPs are translocated through PTEX (Gehde *et al*. 2009; Gruring *et al*. 2012). Furthermore, the PTEX core components are conserved throughout the *Plasmodium* genus indicating a similar export mechanism for all exported proteins and in all *Plasmodium* species. PTEX, and thus protein export, has been shown to be essential for asexual blood stage development in both *P. falciparum* and *P. berghei* (Matthews *et al*. 2013; Matz, Matuschewski and Kooij 2013). However, in gametocytes the function of PTEX is only essential during the first 24–48 hours (stage I/II) of gametocytogenesis. This coincides with the upregulation of GEXPs during early gametocytogenesis and highlights the importance of protein export in this stage.

During further progression of *P. falciparum* gametocytogenesis, the expression levels of PTEX components decrease and their localisation in the PV and PVM becomes less pronounced. In contrast, PTEX components could either not be detected or were not localised to the PV in mature *P. berghei* gametocytes, raising the question whether protein export even occurs at this stage (Matthews *et al*. 2013). In *P. falciparum*, it has not been conclusively shown if protein export still occurs in stage III or later. In fact, the IMC engulfs the entire parasite in stage IV and V gametocytes and could represent a major obstacle for proteins to be trafficked to the parasite plasma membrane (PPM) and beyond.

### Remodelling of host membranes in gametocytes

There are several major changes to the host cell that occur in stage III-V gametocytes, which could be caused by the activation of previously exported effector proteins or by *de novo* export. One of these changes is the deformability switch that occurs during the transition from stage IV to stage V gametocytes. The STEVOR family of exported proteins has been shown to play an important role in mediating host cell rigidity. Whilst still present at the RBC membrane in stage IV, STEVOR is internalised in stage V, potentially contributing to the increase in deformability at this stage (Tiburcio *et al*.2012a; Ramdani *et al*. 2015; Naissant *et al*. 2016). Likewise, changes in the host cytoskeleton network, including spectrin and band 3 (Dearnley *et al*. 2016) may be required for the observed deformability switch during gametocyte maturation.

In asexual *P. falciparum* parasites, Maurer's clefts play an important role in the trafficking of exported proteins within the host cell. Especially, proteins that contain one or more transmembrane domains are temporarily localised to the Maurer's clefts before being further trafficked to the RBC surface (e.g. McMillan *et al*. 2013; Oberli *et al*. 2014). A similar mechanism could exist in gametocytes with effectors being stored at or in the Maurer's clefts and then further trafficked to the site of action when required. Maurer's clefts, originally believed to be injuries to the host cell caused by the attachment of parasites (Mundwiler-Pachlatko and Beck 2013), are generated within hours after invasion of a RBC by *P. falciparum* (Gruring *et al*. 2011). Initially mobile, they are tethered to the RBC membrane and PVM by proteinaceous tethers containing the protein MAHRP2 during the transition from a ring stage parasite to a trophozoite (Hanssen *et al*. 2008; Pachlatko *et al*. 2010; Gruring *et al*. 2011). These tethers have so far only been described in *P. falciparum*, and MAHRP2 is specific to the subgenus *Laverania*, indicating that this mechanism is lineage specific. In *P. falciparum* Maurer's clefts can be detected throughout gametocytogenesis (Dearnley *et al*. 2016). In contrast to trophozoites, Maurer's clefts seem to be mobile in later gametocytes (stage III); however, it is unclear whether they are mobile throughout gametocytogenesis or initially tethered (Dearnley *et al*. 2016). The function of Maurer's clefts tethering is unknown, but it could potentially contribute to its function as a trafficking hub. In *P. falciparum*, a major function of Maurer's clefts is the trafficking of PfEMP1. Disruption of Maurer's clefts architecture by knock-out or truncation of the resident Maurer's cleft protein REX1 prevents the display of PfEMP1 on the RBC surface (Spycher *et al*. 2008; Dixon *et al*. 2011). Whilst REX1 is present at the Maurer's clefts throughout gametocyte development (Dearnley *et al*. 2016), both PfEMP1 and the knob structures that anchor the protein at the RBC membrane are absent in gametocytes (Tiburcio *et al*. 2012b). However, the presence of REX1 indicates that the maintenance of Maurer's clefts architecture throughout gametocytogenesis might be important for transmission. Maurer's clefts or similar structures seem to exist throughout the *Plasmodium* genus, suggesting a conserved function (Aikawa, Miller and Rabbege 1975). There is evidence that they serve similar functions in the trafficking of virulence proteins across species, as the knock-out of two Maurer's cleft resident proteins (MAHRP1, SBP1) results in reduced iRBC receptor binding in both *P. falciparum* and *P. berghei* (De Niz *et al*. 2016). In line with the observed absence of host cell modifications on the iRBC surface of immature gametocytes, binding studies have shown no significant interactions with endothelial cells (Silvestrini *et al*. 2012) or erythroid cells (Neveu *et al*. 2018); however, recent work suggests some binding to mesenchymal stem cells (Messina *et al*. 2018).

Most research into protein export and host cell modifications has been focused on asexual *P. falciparum* stages. The knowledge about iRBC surface antigens in gametocytes will be of great interest for the development of transmission blocking vaccines. Furthermore, understanding the mechanisms of protein export and antigen display during gametocyte development will expand our knowledge about parasite biology and could explain some of the differences between *Plasmodium* species.

### Concluding remarks

Mature gametocytes were first discovered by Alphonse Laveran in the blood of an Algerian soldier more than 130 years ago (Laveran 1881), yet their biology remains elusive to this day. Recent efforts in malaria elimination and eradication have resulted in renewed interest in basic and translational aspects of malaria transmission.

In the past 5 years a series of technical breakthroughs in the malaria field have made the study of parasites and in particular of gametocytes more tractable—(i) forward genetic screens in *P. berghei* and *P. falciparum* (Bushell *et al*. 2017; Zhang *et al*. 2018) allow genome-wide investigation of gene function, which is particularly interesting for facultative processes such as gametocytogenesis; (ii) reproducible protocols for the efficient production of gametocytes (Brancucci *et al*. 2015; Brancucci *et al*. 2017; Filarsky *et al*. 2018) allow for systematic and synchronous investigation of gametocyte development; (iii) successful isolation of subpopulations of cells for transcriptomics (Pelle *et al*. 2015) and proteomics (Khan *et al*. 2005; Silvestrini *et al*. 2010); (iv) development of various inducible expression systems (de Koning-Ward, Gilson and Crabb 2015) and (v) high throughput single cell approaches (Poran *et al*. 2017; Brancucci *et al*. 2018; Reid *et al*. 2018) provide a template for the analysis of rare populations *in vitro* and *in vivo* at scale. These and other emerging tools will enable comparative transcriptomic, proteomic and metabolomic analyses of various parasite species and stages *in vitro* and *in vivo*. At the same time the continuous development in imaging technologies, including super resolution and high content imaging approaches, provide the corresponding phenotypic data at increasing resolution in time and space.

We anticipate that the systematic application of these tools to the study of gametocytogenesis will unravel some of the major questions in the field as follows: (i) how are external signals such as LysoPC translated in the cell to regulate sexual commitment? (ii) how and when is gender determined? (iii) what is the function of IMC, cytoskeleton and parasite surface antigens during gametocyte development *in vivo*? (iv) what is the regulatory program governing gametocyte maturation and mosquito infectivity? (v) what are the functional differences between gametocytes of different lineages? Closing these knowledge gaps may translate into novel opportunities to block the transmission cycle of this deadly human parasite.

## Supplementary Material

fuz010_Supplement_Table
