# Corrigendum to: Revisiting gametocyte biology in malaria parasites

**DOI:** 10.1093/femsre/fuz020

**Published:** 2019-08-06

This manuscript has been amended to correct the author list. This correction is to reflect that Priscilla Ngotho and Alexandra Blancke Soares are co-first authors.

